# Programmable µChopper Device with On-Chip Droplet Mergers for Continuous Assay Calibration

**DOI:** 10.3390/mi11060620

**Published:** 2020-06-25

**Authors:** Nan Shi, Christopher J. Easley

**Affiliations:** Department of Chemistry and Biochemistry, Auburn University, Auburn, AL 36849, USA; nzs0063@auburn.edu

**Keywords:** droplets, lock-in detection, real-time calibration, homogeneous immunoassay, on-chip mergers, pneumatic valves, programmable droplet formation

## Abstract

While droplet-based microfluidics is a powerful technique with transformative applications, most devices are passively operated and thus have limited real-time control over droplet contents. In this report, an automated droplet-based microfluidic device with pneumatic pumps and salt water electrodes was developed to generate and coalesce up to six aqueous-in-oil droplets (2.77 nL each). Custom control software combined six droplets drawn from any of four inlet reservoirs. Using our μChopper method for lock-in fluorescence detection, we first accomplished continuous linear calibration and quantified an unknown sample. Analyte-independent signal drifts and even an abrupt decrease in excitation light intensity were corrected in real-time. The system was then validated with homogeneous insulin immunoassays that showed a nonlinear response. On-chip droplet merging with antibody-oligonucleotide (Ab-oligo) probes, insulin standards, and buffer permitted the real-time calibration and correction of large signal drifts. Full calibrations (LOD_conc_ = 2 ng mL^−1^ = 300 pM; LOD_amt_ = 5 amol) required <1 min with merely 13.85 nL of Ab-oligo reagents, giving cost-savings 160-fold over the standard well-plate format while also automating the workflow. This proof-of-concept device—effectively a microfluidic digital-to-analog converter—is readily scalable to more droplets, and it is well-suited for the real-time automation of bioassays that call for expensive reagents.

## 1. Introduction

Droplet-based microfluidics is an important subcategory of microfluidic technology. In these types of micro-devices, small droplets are generated and viewed as individual reactors, and they provide powerful platforms for confining samples to small volumes for subsequent manipulation, reaction, and analysis [1]. In the last decade, droplet microfluidics has been widely used in a broad range of biochemical fields, such as nucleic acid/molecule analysis [2,3], drug delivery [4], cell-to-cell communication [5], cell screening [6], tissue analysis [7,8,9], and so on. To ensure constant and predictable outcomes in these applications, it is essential to generate highly uniform droplet volumes [10,11,12], and researchers have developed various methods to do so.

Microfluidic droplet formation techniques can be divided into two categories: passive and active. High throughput droplet generation is much simpler and faster to achieve with passive methods, an obvious advantage in applications that require enormous experimental throughput [13]. By contrast, a major benefit of active droplet generation is its higher flexibility in droplet volume and production rate [14]. Because the vast majority of biochemical reactions and analyses require multiplexed reagents, multiple timed steps, and often multiple conditions (temperature, pH, ionic strength, etc.), tools that allow for a precise control of droplets on demand are becoming increasingly important. Significant efforts have been focused on active droplet formation using various approaches such as electric, magnetic, thermal, and mechanical control [15,16,17,18]. Considering the exquisite level of control that they provide, on-chip pneumatic valves [19] have been demonstrated as important players that provide an active, programmable droplet generation with high precision [7,9,15,20,21,22]. 

To improve programmability and precision, our laboratory has moved from passive droplet formation [11,12], to active fluidic resistors [21], to the gating of fluids with single pneumatic valves [8,22], and finally to on-chip valve-based pumps [7,9]. During this time, we revealed one less obvious benefit of active control: the ability to precisely control the frequency and phase of droplets, lock in the photodetector to that signal, and greatly reduce the detection limits—an approach we refer to as the μChopper [8,12,22]. With a control bandwidth of ±0.04 Hz using gating valves, the fluorescence detection limits were reduced more than 50-fold using simple microscope detection optics, and even single-cell fatty acid uptake was quantifiable in droplets [8]. An improved iteration of the µChopper with six aqueous input channels enabled several analytical modes to be programmed automatically, such as real-time continuous calibration, standard addition, and a mixed mode [22]. Despite these benefits, there remains a drawback with respect to the workflow in this type of microsystem. Reagents for multi-step or timed reactions must be manually pre-mixed and transported to the input micro-reservoirs, increasing the bench time and potential operator errors. The logical step is to add on-chip reagent mixing or to incorporate programmable droplet mergers. 

The Ismagilov group and others have successfully initiated the mixing of reagents at the droplet forming structure [7,23,24,25], which can start reactions at a predictable position and provide control over timing. However, several issues limit the accuracy and preclude the universal application of this approach. First, inconsistent flow rates of solutions from individual aqueous channels can lead to fluctuating reagent volume ratios and significantly affect assay outcomes. Second, it is difficult to precisely and arbitrarily change the volume ratio of reagents, meaning that new channel designs will be needed for even minor adjustments. Several techniques to coalesce neighboring droplets were introduced to avoid these issues, such as hydrodynamic, magnetic, electric, or acoustic coalescence [26,27,28,29,30]. Among these, electrocoalescence has been the most widely used in droplet microfluidics by merging adjacent droplets with an alternating current (AC) electric field applied to nearby electrodes on the device. The development of in-channel “salt water electrodes” by the Abate group, where high-concentration salts can replace metal solder, has made this approach even more accessible [28].

Considering the benefits of pneumatically controlled droplet generation and electrocoalescence, here we have integrated our µChopper approach with active valve-based pumps and salt-water electrodes for the first time. This approach permits the fully automated, on-demand production and merging of several types of droplets in a programmable way. In this proof-of-concept work, we apply the device to the real-time, continuous calibration of fluorescent labels, and then we validate the system for the continuous calibration of a homogeneous insulin immunoassay that exhibits a nonlinear response. With the significant savings in reagent use, assay cost, and user time that are incurred, this device provides a novel means to carry out economical measurements with precious reagents in a static or real-time manner.

## 2. Materials and Methods

### 2.1. Materials and Equipment

All materials and equipment were obtained from sources within the USA. Buffers were prepared using deionized water filtered with a Barnstead MicroPure Water Purification system (ThermoFisher Scientific, Waltham, MA, USA). Citric acid, sodium phosphate, and sodium chloride were obtained from Millipore Sigma (Burlington, MA, USA). Polydimethylsiloxane (PDMS) precursors, SYLGARD 184 silicone elastomer base, and curing agent were purchased from Dow Corning (Midland, MI, USA). The silicon wafers were acquired from the Polishing Corporation of America (Santa Clara, CA, USA). Negative photoresists (SU-8 2015 and SU-8 2050) and SU-8 developer were purchased from MicroChem (Westborough, MA, USA). Positive photoresist (AZ 40XT-11D) and AZ 300 MIF developer were obtained from AZ Electronic Materials USA (Somerville, NJ, USA). Fluorescein was purchased from Alfa Aesar (Ward Hill, MA, USA), and bovine serum albumin (BSA) was purchased from VWR (West Chester, PA, USA). Human Insulin FRET-PINCER Assay Kits were obtained from Mediomics, LLC (Saint Louis, MO, USA). Pico-Surf (2% in Novec 7500 oil), a perfluorocarbon surfactant, was purchased from Dolomite Microfluidics (Norwell, MA, USA) for stabilizing droplets against unwanted coalescence and to provide biocompatible surfaces within the droplets. Novec 7500 Engineered Fluid (HFE 7500) was acquired from 3M (St. Paul, MN, USA). 

**Figure 1:**
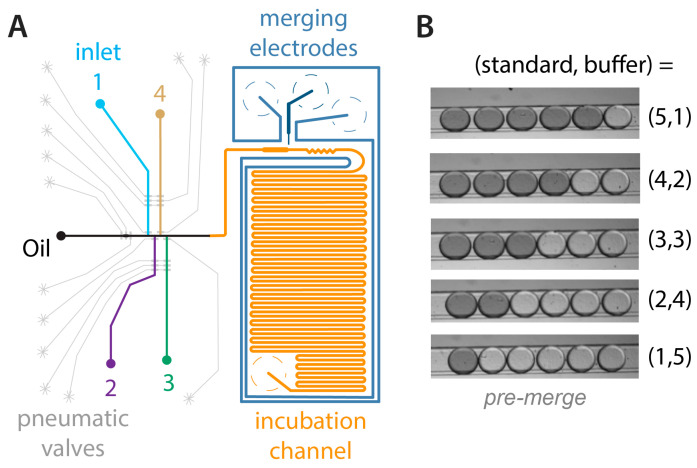
Microdevice design and operation. (**A**) Inlet aqueous reservoirs (1–4, colored) and one oil reservoir (black) were sampled by computer-controlled pumps based on pneumatic valves (light gray). Merging electrodes (dark blue) facilitated droplet coalescence in the widened merging region (orange), merged droplets were mixed in a zig-zag channel, and then assays were incubated in a long delay channel (orange) if needed prior to optical detection. (**B**) In this example, five ratios of standard mimics (dark) and buffer (transparent) were programmed on demand, then merged downstream. Images show the droplet groups prior to merging (see Videos S1 and S2).

A high voltage amplifier (Model 2220) was purchased from Trek, Inc. (Lockport, NY, USA) and used for droplet merging. Fluorescence excitation and emission were accomplished using a Nikon Ti-E inverted fluorescence microscope (40X objective, 0.75 NA; Nikon Instruments Inc., Melville, NY, USA) interfaced to a CCD camera (CoolSnap HQ2; Photometrics Scientific, Tucson, AZ, USA). Fluorescence images were acquired by focusing on a chosen region of interest in the incubation channel (Figure 1A) and collecting at 100 frames s^−1^ through the green fluorescence filter cube (λ_ex_ = 470 ± 20 nm, λ_em_ = 525 ± 25 nm).

### 2.2. Microfluidic Master Wafer Fabrication

Two master wafers for templating liquid channels and pneumatic control channels were fabricated using standard photolithography as described previously [9]. Channel layouts were designed in Adobe Illustrator software, and plastic film photomasks were printed at Fineline Imaging (Colorado Springs, CO, USA) at a 50,800 dpi resolution. For the pneumatic control channel layer, a ~20 μm layer of SU-2015 was spin-coated on the silicon wafer, which had been washed by 1 M H_2_SO_4_ and water in advance. The wafer was soft-baked at 95 °C or 5 min, after which ultraviolet (UV) light exposure for 2 min was accomplished on an in-house built UV lithography light exposure unit [31]. Finally, the wafer was developed for 5 min in SU-8 developer solution after a 5-min hard bake on a hot plate at 115 °C. The fluidic layer wafer was fabricated in a two-step protocol with both negative and positive photoresists, respectively. First, a 60-μm layer of SU-8 2050 was spun onto the pretreated silicon wafer, and the wafer was soft baked at 95 °C for 7 min. UV exposure with the first photomask was carried out for 90 s, hard baking was applied at 95 °C for 6 min, and then the SU-8 developer was applied. Next, a 40-μm layer of AZ 40 XT was spun onto the wafer at room temperature. The wafer was baked at 115 °C for 5 min, cooled to room temperature passively, and the second photomask was aligned over the wafer then exposed to UV light. A final hard bake at 115 °C was applied for 1.5 min, AZ developer was applied, and the AZ portion of the wafer was annealed to allow templating of rounded channel cross-sections by baking at 120 °C for 10 min.

### 2.3. Microchip Fabrication 

After degassing under vacuum, 36 g of well-mixed PDMS precursor mixture (5:1 ratio, monomer: curing agent) was poured onto the flow channel patterned silicon wafer in an aluminum foil boat. Again after degassing, 5.12 g of PDMS precursor mixture (15:1 ratio, monomer:curing agent) was spin-coated onto the control layer at 2100 rpm for 45 s, creating a layer of ~40 μm thickness. Both the fluid layer and control layer were baked at 65 °C for 30 min in an oven. The flow channel layer was then cut to shape, then aligned and mated to the valve channel layer. The two mated layers were baked in an oven at 65 °C overnight to facilitate permanent bonding. The PDMS was peeled from the wafer, diced into individual devices, access reservoirs were punched, and the surfaces were washed with methanol and dried with N_2_ gas. Each device was then irreversibly bonded to a glass slide by plasma oxidization (Harrick Plasma; Ithaca, NY, USA). The assembled microfluidic devices were finally thermally aged at 65 °C overnight to limit uncured PDMS monomer leakage, and these devices were then ready to use.

### 2.4. Flow Control and Droplet Generation 

For generating droplets on demand, a total of 19 pneumatic push-up valves on the microfluidic chip were programmatically controlled by an in-house written LabVIEW application which was interfaced to a custom manifold of solenoid switches (LHDA0533115H; the Lee Company, Westbrook, CT, USA) using a multifunction data acquisition system (PCI-6259, National Instruments). These solenoid valves were actuated by 5 V signals to controllably switch a pressurized nitrogen supply (25 psi), and only 13 solenoids were needed due to redundancy in operating some valves in the peristaltic pumps. For the periodic rinsing of the microdevice, the outlet could also be connected to a hand-held 100-mL syringe via Tygon tubing (0.02″ I.D. X 0.06″ O.D.; Cole-Parmer, Vernon Hills, IL, USA) to allow a small vacuum to be applied.

Droplets were generated with three-valve peristaltic pumps as described previously [7,9]. Oil segments were pumped in between each aqueous segment at a T-junction channel to form aqueous-in-oil droplets, and this formation was precisely controlled in an automated fashion using LabVIEW (Figure S1).

### 2.5. Programmable Merging of Droplets with Salt Water Electrodes 

Droplets were merged with a 10 kHz alternating current (AC) signal of 500 V applied to nearby channels (“merging electrodes”) filled with 5 M NaCl. The high voltage amplifier (Trek, Inc., Lockport, NY, USA; Model 2220) was controlled using an in-house written LabVIEW application. The merging region was widened when compared to incoming and outgoing channels to facilitate a slower migration and improved droplet contact for merging. This methodology was described in more detail by Sciambi and Abate [28].

User-defined time and channel programs for automatically building real-time five-point calibrations within sequentially merged droplets were preloaded into an in-house written LabVIEW application (Figure S2). Briefly, sequential groups of six droplets (2.77 nL each) were formed and separated in space to prevent group-to-group merging, and these six droplets were merged (16.6 nL in each larger droplet) using electrocoalescence downstream. As such, two types of oil segments were programmed: very short oil segments to keep droplets in the same group as close as possible, and longer oil segments to partition the sequential droplet groups. As discussed above, 84 possible solution combinations could be programmed into the finally merged droplet under the conditions investigated here. When applicable, the concentrations of an unknown sample could be determined in real time using continuous calibration curves, and signal drifts were corrected using the µChopper concept.

## 3. Results and Discussion

### 3.1. Microfluidic Device Design and Operation

As shown in Figure 1A, the microdevice was defined by several regions: (1) four different aqueous inlet reservoirs (colored) and an oil inlet reservoir (black); (2) T-junction channels for aqueous-in-oil droplet generation (colored and black); (3) pneumatic control channels (light gray) for automated chip operation through LabVIEW (National Instruments, Austin, TX, USA) ), with some three-valve pumps integrated to improve efficiency; (4) salt water electrodes for droplet coalescence with a high AC voltage (two tones of dark blue); (5) a widened merging region near the salt water electrodes, at the sharpest electric field gradients; (6) a zig-zag channel (orange) for quickly and completely mixing reagents contained in droplets; and (7) a long incubation channel for storing and analyzing target droplets (orange). Regions (1) and (2) were valve-controlled AZ-defined rounded channels of ~40 µm depth, region (3) was SU-8 defined rectangular channels of ~20 µm depth, and regions (4)–(7) were SU-8 defined rectangular channels of ~55 µm depth.

In a typical assay workflow, calibration curves are regularly generated to allow the measurement and calculation of an unknown sample concentration. The conventional method is to quantify sequential standard solutions followed by each sample, then calculate the response curve and quantify samples post-measurement. Particularly when using expensive reagents—such as antibodies, protein standards, enzymes, bioconjugates, etc.—this traditional process not only wastes significant amounts of materials but also increases the workload of operators. To improve the accuracy and efficiency of building standard curves, we recently developed a six-channel µChopper to automatically carry out a continuous calibration mode [22], allowing the real-time determination of the slope, y-intercept, and correlation coefficients, along with unknown quantification. In this report, we improve upon this concept by introducing downstream droplet mergers via electrocoalescence, and we provide an even more precise control using on-chip pneumatic pumps. 

To achieve full automation, we developed the device design in Figure 1A, which allows the programmable generation of droplets in various combinations from any of four input aqueous reservoirs. An example of a programmable calibration is shown in Figure 1B, where droplets containing dye solution (mimicking assay standards) and buffer are generated in various ratios. The images show droplets prior to merging into a single, larger droplet. Videos of programmable droplet formation (Video S1) and downstream merging (Video S2) are provided as supporting information. In this work, the total droplet number was limited due to the size of the coalescence region; however, this number could be increased by simply enlarging the dimensions of this region. With four input reservoirs and the total droplet count fixed at six, this system allowed for 84 possible solution combinations to be programmed into the finally merged droplet (16.6 nL). Notably, if the total droplet count were expanded to be one through six—easily accomplished with this device—there would be 209 possible solution combinations. The upper limit can be extended if the coalescing region is made larger; for example, if 24 total droplets were allowed (six from each reservoir), then 2400 combinations would be accessible.

### 3.2. Microdevice Characterization with Continuous Linear Calibration

**Figure 2:**
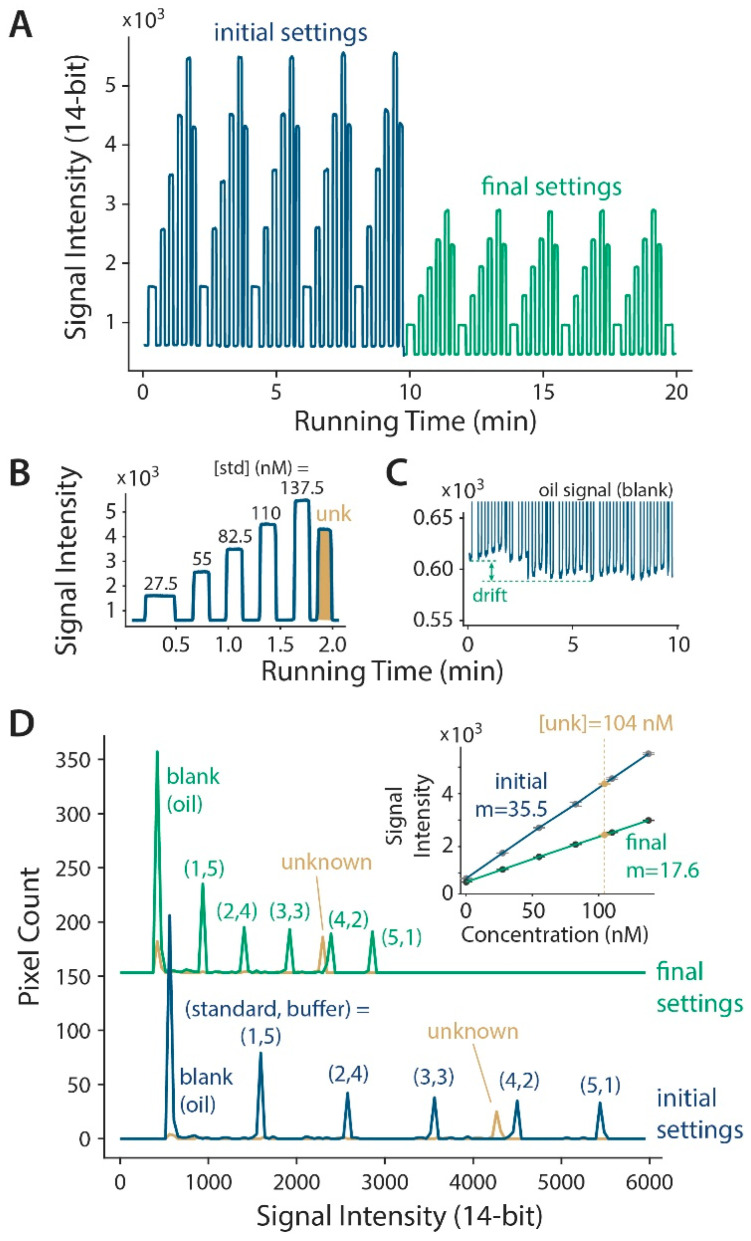
Continuous calibration with automated droplet formation and merging. (**A**) Raw fluorescence emission data shows that the droplet contents were programmable. Data is shown under initial settings at a higher excitation light intensity (blue) and with final settings after decreasing the light (green) in real time. (**B**) A magnified segment of this data, with pulses labeled using final, post-merge concentrations of fluorescein standard. Data from the unknown droplet is shaded in gold. (**C**) Magnified view of the oil signal shows a typical optical system drift that can be corrected using our µChopper method [8,12,22]. (**D**) Histogram analysis reveals the method’s capability for a highly precise control of the droplet contents. The peaks are labeled with the pre-merge, programmed numbers of standard and buffer droplets. The inset shows the linear calibrations under the initial and final settings.

To verify the automation capabilities of our device, fluorescein standard (165 nM), buffer, and an unknown fluorescein sample were loaded into reservoirs #1, #2, and #3, respectively (see Figure 1A). Five calibration standards were formulated by sequentially generating and coalescing groups of six droplets at varying ratios of fluorescein standard and buffer (1-5, 2-4, 3-3, 4-2, 5-1; akin to Figure 1B). For unknown measurements, a single larger-volume droplet was sampled from reservoir #3 and kept separate from the standard droplets. The blue and green traces in Figure 2A show a 20-min record of the raw fluorescence data measured at the incubation channel during the continuous calibration. To challenge the system during the continuous calibration, the excitation light was changed from a higher (initial settings) to a lower intensity (final settings). The unknown droplet’s signal decreased by ~50% following this light intensity change. Since the signal from all of the calibration standards also decreased by the same proportion (~50%), the system allowed an accurate calibration to be maintained despite the challenge. Figure 2B shows a magnified view of the signal from one group of calibration standards and an unknown, and Figure 2C highlights the detector-dependent, low-frequency drift (noise) that can be corrected using the lock-in-based µChopper method.

As shown in Figure 2D, the programmable droplet formation and merging enables a high-precision control over the final droplet composition. Essentially, this device operates as a microfluidic digital-to-analog converter, albeit at a relatively low resolution. The intensity histograms show that the fluorescence intensities were accurately controlled by the programmed ratios of the standard and buffer droplets, from (1,5) to (5,1). The initial settings (blue) showed that the unknown fluorescence was nearly as high as the (4,2) droplet, and after the light intensity challenge the final settings (green) showed the same order and position, just at a lower intensity. The inset calibration curves in Figure 2D show that the light intensity decrease mainly affected the calibration slope, while the unknown was determined to be at a concentration of 104 nM, independent of the excitation light intensity. These data point to a major advantage of continuous calibration, where the system can automatically adjust to drastic changes in the environmental conditions.

### 3.3. Unique Data Reshaping Using MATLAB Code

**Figure 3:**
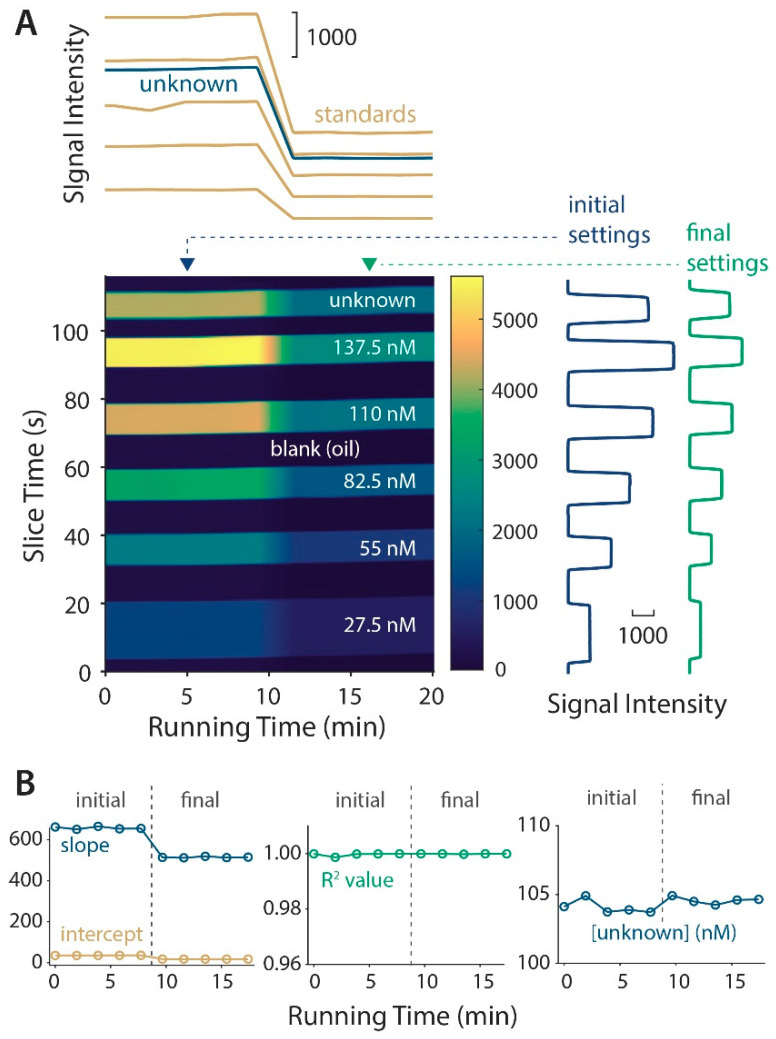
Data reshaping allowed for a unique visual inspection of the system, enabled by a precise droplet control with valves. (**A**) The raw data vector over time was reshaped into an image array using custom a MATLAB code, and image re-slicing permitted temporal tracking of each type of droplet (above) or original data recovery (right). (**B**) The system responded to the light intensity decrease by adjusting the calibration parameters, while the fit linearity and unknown determination were essentially unaffected.

Since on-chip valves provide highly precise and programmable droplet intensities with repeatable timing, we surmised that the raw data could be readily reshaped into a more easily readable image format. Using a custom MATLAB (MathWorks, Natick, MA, USA) code (see Supporting Information), the raw data from Figure 2A was sliced into segments representing the repeating groups of larger droplets (116-second slices), and these slices were restacked over the running time of ~20 min and presented as the image in Figure 3A. The image intensity represents the 14-bit camera signal intensity using a custom colormap depicted in the legend. The five stripes of intense signal in this image represent a tracked intensity of each type of merged droplet, and the darker regions are the larger oil segments that separate them. For example, the blue stripe near the bottom of the image is a reshaping of data from multiple 27.5 nM standard droplets, where (standard,buffer) = (1,5), and this image allowed a facile tracking of their intensities over time. Indeed, the re-slicing of the image horizontally (shown above the image) gave the time traces of each standard (gold) and the unknown (blue). Again, with the change in light intensity, it is obvious that all calibration standards shifted along with the unknown to maintain calibration integrity. Conversely, by re-slicing the image vertically, the original data can be recovered, as shown at the right during both the initial (blue) and final (green) settings. This novel data reshaping approach is well-suited to an automated, droplet-based continuous calibration, and it was enabled by the precision of the valve-based control. It should be noted that a lock-in analysis was not yet applied to the data in this reshaped image, but further development of image analysis algorithms in the future will allow a lock-in analysis directly from these types of images.

This analysis gives a unique, visual means to showcase the system’s ability to respond to environmental changes. Following the lock-in analysis, Figure 3B depicts the system’s response to the challenge, where the major adjustment was a decrease in the slope (blue) of the linear calibration curve and a small change in the y-intercept (gold). The R^2^ value (green) remained at a high level near 1.00 over the course of the sampling, and the unknown determination was steady at 104 nM despite the change in the excitation light intensity (blue data on the right, magnified to a 100–110 nM range).

### 3.4. Continuous Calibration Using a Nonlinear Homogeneous Immunoassay

**Figure 4:**
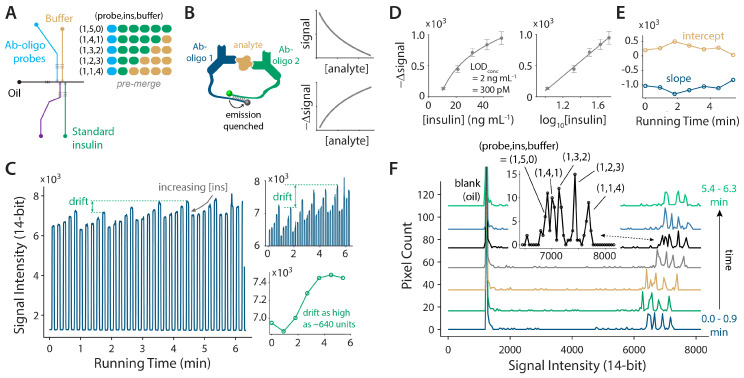
Automated homogeneous immunoassays in nanoliter droplets. (**A**) Device was operated with three inlets to program the pre-merge ratio of Ab-oligo probe, insulin, and buffer droplets. (**B**) Fluorescence-quenching-based homogeneous immunoassay with Ab-oligo probes. The signal quenching is proportional to the analyte concentration with a nonlinear response curve. (**C**) Raw emission data from the automated continuous calibration. The upper inset is a zoomed view of the detector drift, and the lower inset shows that the magnitude of the drift is similar to the overall assay response. (**D**) Lock-in detection with the µChopper method allows for a reliable correction and calibration. The signal change is shown versus [insulin] (left) and log_10_[insulin] (right). LOD_conc_ = 2 ng mL^−1^ = 300 pM, while LOD_amt_ = 5 amol. (**E**) The continuous linear calibration parameters versus log_10_[insulin] show the slope and y-intercept to be responsive to significant detector drifts. (**F**) The intensity histograms show that the assay responses over the 10–50 ng mL^−1^ insulin range were closely clustered, and drift could also be observed. The calibration standards followed the drift, giving reliable calibrations over time as in part (**D**,**E**).

Finally, we tested the performance of this droplet-based system using a more complex assay response. Using antibody-oligonucleotide conjugates (Ab-oligos) as probes (Figure 4B), where deoxyribonucleic acid (DNA) arms are labeled with a fluorophore and quencher, it is possible to quantify a protein analyte with a high specificity through a mix-and-read workflow [7,32,33] that is ideal for detection within droplets. However, the recovery of the sometimes small, unamplified signal changes can be challenging, particularly in the biologically relevant ranges for a hormone such as insulin (low ng mL^−1^; pM to nM). We previously showed that our µChopper method provides a key enhancement to enable homogeneous immunoassays within droplets [8], and the combined techniques even allowed high-resolution sampling of insulin secretion from single pancreatic islets [7]. The disadvantages in these devices were that the mixing ratios of Ab-oligos and the sample were device-dependent, the assay timing was restricted by the flow rates of the on-chip pumps, and the calibrations had to be carried out before or after experiments in a serial fashion. Here (Figure 4A), we show that by forming aqueous-in-oil droplets of Ab-oligo probes (blue), insulin analyte (green), and buffer (gold) in a programmable way and then merging them downstream with integrated electrocoalescence, all of these aforementioned problems can be solved.

By virtue of valve-based automation and downstream droplet merging, precious Ab-oligo reagents did not need to be diluted, premixed, or incubated with samples. For each measurement, a single drop of the stock Ab-oligo was sampled from inlet #1 (blue) and grouped with varying numbers of insulin (#3, green) and buffer (#4, gold) droplets (Figure 4A), and these six droplets were merged, mixed, and incubated downstream to allow for continuous calibration. The raw emission data in Figure 4C shows that the quenching within droplets was proportional to [insulin]. Also shown is the magnitude of the detector drift (two inset plots), which becomes highly significant compared to the signal changes in these homogeneous immunoassays at low analyte concentrations. In fact, the drift as high as ~640 intensity units was similar to the overall assay change for the full calibration range. Using the µChopper approach [8,12,22], these drifts were negated to give consistent calibration results over the entire experiment. The average curves are shown in Figure 4D, while the real-time curve parameters (linear fits versus log_10_[insulin]) are shown in Figure 4E. Continuous calibration allowed drifts and/or environmental changes to be negated, where the system continuously adjusted by modifying the slope and y-intercept. The concentration limit of detection (LOD_conc_) was 2 ng mL^−1^ (300 pM), while the number of moles that were detectable (LOD_amt_) was 5 amol (5 × 10^−18^ mol). This LOD is the best achieved to date for homogeneous insulin immunoassays using droplet-based microfluidics [7,8]. These data prove that the programmable device can give highly precise amounts of probe, calibration standards, and buffer—a significant improvement compared to the laminar flow sampling method in prior devices, where chip-to-chip variations were significant.

The histograms shown in Figure 4F highlight some challenges that may arise using nonlinear, homogeneous immunoassays at low concentration ranges compared to simple direct fluorescence (as shown in Figure 2D). Calibration intensities were clustered together between 6000 and 8000 intensity units, and the drift could be readily observed. The inset magnified plot shows that the three highest [insulin] values—made with droplet ratios (1,5,0), (1,4,1), and (1,3,2)—were just barely resolved under these conditions. Fortunately, the µChopper method can compensate for these effects [8,12,22]. Overall, the data in Figure 4 show that automated sampling and downstream merging, when combined with lock-in detection, provide a highly reliable way to perform mix-and-read immunoassays in nanoliter droplets. The system can even be applied to the real-time quantification of proteins. These benefits were achieved with minimal user intervention, where the workflow consisted of adding merely three solutions to the inlet reservoirs before starting the system.

Lastly, an additional and noteworthy improvement is reduced cost. Using this device, the total volume used in one five-point calibration for the insulin immunoassay was 83.1 nL (five merged droplets of 16.6 nL). For the most expensive components, Ab-oligo probes, only 13.85 nL was required. Because a five-point calibration in the standard 384-well plate version of the assay requires 2.25 µL of probes, our device reduced the needed volume 160-fold, which translates to an equivalent 160-fold reduction in cost for these precious bioconjugate components.

## 4. Conclusions

A fully automated microchip was introduced to precisely and rapidly form droplets of sequential calibration standards, allowing quantitative analyte measurements in the nanoliter range and in real time. The key novelty of this device was the integration of valve-based automation, on-chip droplet electrocoalescence, and µChopper data analysis. The user workflow was minimized to a few solution transfer steps at the beginning of the experiment, and cost reductions of more than two orders of magnitude (160-fold) were realized with homogeneous insulin immunoassays. Furthermore, full calibrations required <1 min, and this system also posted the lowest LOD achieved to date using droplet-based homogeneous immunoassays (insulin LOD_conc_ = 2 ng mL^−1^ = 300 pM; LOD_amt_ = 5 amol). The highly precise, programmable control also permitted unique data reshaping into images, with which lock-in detection or continuous referencing should be feasible through image analysis improvements.

Of course, some challenges remain with this system, which could be addressed in future work. To accommodate the serial sampling of multiple droplets and the formation of groups of calibration standards, the overall sample flow rate using on-chip pumps was lowered, causing about a one-order-of-magnitude loss in the temporal resolution of the sampling. Thus, the method timing is not yet competitive with our own state-of-the-art sampling resolution of 3.5 s [9]. While this issue is a cost of automation that is partially offset by the benefits, it could likely be improved by using gating valves [8] instead of full three-valve pumps. Finally, increases in the size or volume of the merging region would allow more droplets to be merged (>6). Changing this feature, along with increasing the input reservoir number, would exponentially increase the number of possible solution combinations, perhaps making it more palpable to refer to the chip as a microfluidic digital-to-analog converter.

## Supplementary Materials

The following are available online at https://www.mdpi.com/2072-666X/11/6/620/s1, Figure S1: Automated µChopper device design and timing during operation, Figure S2: Programmatic flow chart of the LabVIEW application, Text: MATLAB code for reshaping data into an image, Video S1: “droplet generation”, Video S2: “six merge”.
