# Supplementary material for: Programmable µChopper Device with On-Chip Droplet Mergers for Continuous Assay Calibration
Source: Micromachines (Basel). 2020 Jun 25;11(6):620. doi: 10.3390/mi11060620 (PMC7344876; doi:10.3390/mi11060620)
Supplement: Supplementary file 1 [file micromachines-11-00620-s001.zip › micromachines-842447-supple-for final/micromachines-842447-supple-for final.docx]

Supporting Information

Programmable µChopper Device with On-Chip Droplet Mergers for Continuous Assay Calibration

Nan Shi and Christopher J. Easley

Supporting Information (SI) Contents:

Page S-2: Automated µChopper device design and timing during operation, Figure S-1.

Page S-3: Programmatic flow chart of the LabVIEW application for automatically generating real-time continuous calibration, Figure S-2.

Page S-4: Custom MATLAB code for reshaping raw droplet data into an image.


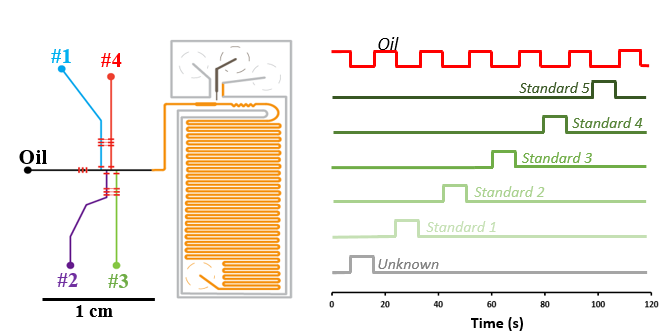


**Figure S1.** Automated µChopper device design and timing during operation. **(A)** The schematic layout shows rounded AZ channel contained oil channel (black) and four aqueous channels (#1, #2, #3, #4), squared SU-8 channels including incubation channel (orange) , salt water electrode (dark gray) , moat channels (light gray) and pneumatic control channels (red). **(B)** The temporal program for one full cycle of programmable continuous calibration.


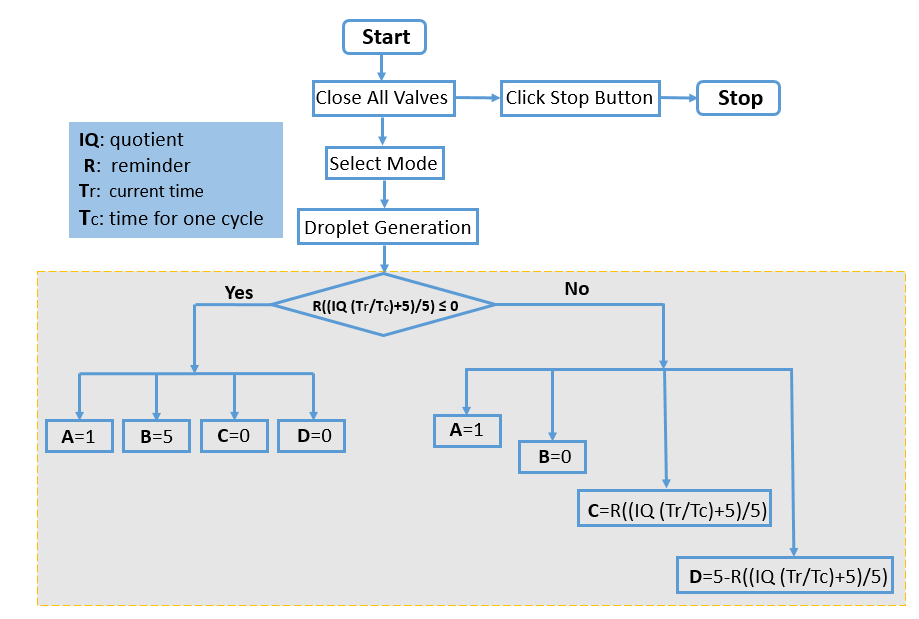


**Figure S2.** Programmatic flow chart of the LabVIEW application for automatically generating real-time continuous calibration. The total number of droplets for merging in a group was restricted to be six. A, B, C, and D represent the droplet numbers generated from four different aqueous channels, respectively.

Code for Reshaping Data into an Image

Below is the custom code developed in MATLAB, saved as “DropTrackSimple.m”. With this code, raw droplet fluorescence emission data from **Figure 2A** in the main text was sliced into segments representing the repeating groups of larger droplets (116-second slices), and these slices were restacked over the running time of ~20 min and presented as the image in **Figure 3A** (main text).

function [DropImage] = DropTrackSimple(data, window)

%{

Data analysis for automated droplet device

INPUTS:

data = text data with two columns, the first being frame number or time,

and the second being the raw fluorescence intensity

window = size of window to slice and synchronize data (in frame numbers)

OUTPUTS:

DropImage = final image with x-axis representing a "droplet group number"

Coded by Easley laboratory, Auburn University, Auburn, AL, USA

**Updated June 4, 2020**

%}

clf

S = zeros(5,2);

dataRaw = data(:,2);

dataSum = zeros(size(dataRaw));

% Apply windowed smooth filter to raw data

SmoothWin = 13; % width of window

for i = (-floor(SmoothWin/2):floor(SmoothWin/2))

dataSum = dataSum + circshift(dataRaw,i);

end

dataSmooth = dataSum/SmoothWin;

data2(:,1) = dataSmooth;

% Reshape the data into an image using the 'window' input for slicing

for j = 1:(size(data2,1))

DropImage(mod(j,window)+1,ceil(j/window))=dataRaw(j,1);

end

% Display reshaped data as a countour image

figure(1);

contourf(DropImage);

| 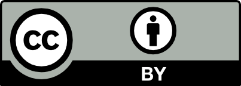 | © 2020 by the authors. Submitted for possible open access publication under the terms and conditions of the Creative Commons Attribution (CC BY) license (http://creativecommons.org/licenses/by/4.0/). |
| --- | --- |
